# Supplementary material for: Dissipation, Metabolism, Accumulation, Processing and Risk Assessment of Fluopyram and Trifloxystrobin in Cucumbers and Cowpeas from Cultivation to Consumption
Source: Foods. 2023 May 22;12(10):2082. doi: 10.3390/foods12102082 (PMC10217671; doi:10.3390/foods12102082)
Supplement: Supplementary file 1 [file foods-12-02082-s001.zip › foods-2317784-supplementary.pdf]

Dissipation, Metabolism, Accumulation, Processing and Risk Assessment of Fluopyram and Trifloxystrobin in Cucumbers and Cowpeas from Cultivation to Consumption

Kai Cui [1], Shuai Guan [1], Jingyun Liang [1], Liping Fang [1], Ruiyan Ding [1], Jian Wang [1], Teng Li [1], Zhan Dong [1,*], Xiaohu Wu [2] and Yongquan Zheng [2]

[1] Institute of Quality Standard and Testing Technology for Agro-Products, Shandong Academy of Agricultural Sciences, Shandong Provincial Key Laboratory of Test Technology on Food Quality and Safety, Jinan 250100, China

[2] Institute of Plant Protection, Chinese Academy of Agricultural Sciences, Beijing 100193, China

*Correspondence: zhandongsaas@163.com

Table S1. Retention time and MRM parameters of FLU and TRI and their metabolites FLB and TRA for HPLC-MS/MS detection.

Table S2. Detailed parameters/exposure factors for human health risk assessment.

Table S3. The calibration regression equation, R^2, ME, and LOQ for FLU and TRI and their metabolites FLB and TRA in different matrices.

Table S4. Residue levels of FLU and TRI and their metabolites FLB and TRA in cucumbers and cowpeas for the different processing operations.

Table S5. Total FLU and TRI residues (expressed as FLU_{sum} and TRI_{sum}) at different sampling intervals and MRLs for different countries.

Table S1. Retention time and MRM parameters of FLU and TRI and their metabolites FLB and TRA for HPLC-MS/MS detection.

Pesticides	Chemical formula	Retention time (min)	Quantitative ion (m/z)	Qualitative ion (m/z)	Fragmentor (V)	CE (V) Quan/qual
FLU	$C_{16}H_{11}ClF_6N_2O$	2.951	397/173	397/207.9	166	40/20
FLB	$C_8H_6F_3NO$	1.942	190/130	190/102		20/35
TRI	$C_{20}H_{19}F_3N_2O_4$	3.297	409.14/186	409.14/145		20/60
TRA	$C_{19}H_{17}F_3N_2O_4$	3.038	395/186	395/148		19/11

Table S2. Detailed parameters/exposure factors for human health risk assessment.

Exposure factors	Unit	Adults	Children
F (cucumber)	g/d	32.10	32.10
F (cowpea)	g/d	7.22	7.22
LP (cucumber)	g/d	424.69	212.11
LP (cowpea)	g/d	507.58	203.31
bw	kg	53.23	16.14
v		3	
ADI (FLU)	μg/kg bw/d	10	
ADI (TRI)	μg/kg bw/d	40	
ARfD (FLU)	μg/kg bw/d	500	
ARfD (TRI)	μg/kg bw/d	-	

Table S3. The calibration regression equation, R^2, ME and LOQ for FLU and TRI and their metabolites FLB and TRA in different matrices.

Pesticides	Matrix	Regression equation	R2	ME(%)	LOQ (μg/kg)
FLU	acetonitrile	y = 9427.21 x + 88415.52	0.9976	-	1
	cucumber	y = 8822.94 x + 17101.50	0.9994	-6.41	1
	boiling cucumber	y = 9199.76 x + 15802.46	0.9993	-2.41	1
	pickling cucumber	y = 8816.14 x + 6850.50	0.9994	-6.48	1
	stir-frying cucumber	y = 9219.29 x + 9797.88	0.9993	-2.21	1
	cucumber skin	y = 9407.08 x + 2645.41	0.9970	-0.21	1
	cucumber pulp	y = 10935.16 x + 7981.78	1	16.00	1
	cowpea	y = 6213.87 x + 4086.91	0.9996	-34.09	1
	boiling cowpea	y = 5556.25 x + 2626.16	0.9996	-41.06	1
	pickling cowpea	y = 6198.98 x + 4771.19	0.9994	-34.24	1
	stir-frying cowpea	y = 5582.53 x + 3024.49	0.9992	-40.78	1
FLB	acetonitrile	y = 2725.10 x + 3139.91	0.9995	-	1
	cucumber	y = 2458.89 x + 1661.14	0.9996	-9.77	1
	boiling cucumber	y = 2478.55 x + 940.91	0.9995	-9.05	1
	pickling cucumber	y = 2428.31 x + 1967.05	0.9994	-10.89	1
	stir-frying cucumber	y = 2454.34 x + 1239.23	0.9994	-9.94	1
	cucumber skin	y = 2422.60 x - 6425.37	0.9969	-11.10	1
	cucumber pulp	y = 2652.23 x + 227.79	0.9999	-2.67	1
	cowpea	y = 1940.19 x + 1698.81	0.9995	-28.80	1
	boiling cowpea	y = 1839.92 x + 1636.48	0.9992	-32.48	1
	pickling cowpea	y = 1953.22 x + 2241.30	0.9994	-28.32	1
	stir-frying cowpea	y = 1850.11 x + 2407.90	0.9990	-32.11	1
TRI	acetonitrile	y = 16904.44 x + 31997.16	0.9943	-	1
	cucumber	y = 9351.69 x + 15165.34	0.9996	-44.68	1
	boiling cucumber	y = 10790.52 x + 11351.66	0.9996	-36.17	1
	pickling cucumber	y = 9090.54 x + 11264.65	0.9994	-46.22	1
	stir-frying cucumber	y = 10545.37 x + 8142.09	0.9995	-37.62	1
	cucumber skin	y = 9712.44 x - 14278.99	0.9979	-42.55	1
	cucumber pulp	y = 13228.03 x + 1837.24	1	-21.75	1
	cowpea	y = 3780.36 x + 11285.26	0.9979	-77.64	1
	boiling cowpea	y = 3709.41 x + 3434.91	0.9989	-78.06	1
	pickling cowpea	y = 3480.58 x + 6978.70	0.9980	-79.41	1
	stir-frying cowpea	y = 3641.20 x - 1791.26	0.9985	-78.46	1
TRA	acetonitrile	y = 6017.56 x - 2615.56	0.9997	-	1
	cucumber	y = 6275.75 x + 1738.30	1	4.29	1
	boiling cucumber	y = 6506.48 x - 390.80	0.9997	8.12	1
	pickling cucumber	y = 6201.46 x + 967.47	1	3.06	1
	stir-frying cucumber	y = 6429.20 x - 91.23	0.9999	6.84	1
	cucumber skin	y = 4753.95 x - 12121.17	0.9970	-21.00	1

	cucumber pulp	y = 5210.86 x - 3220.04	0.9998	-13.41	1
	cowpea	y = 4403.92 x + 4941.56	0.9997	-26.82	1
	boiling cowpea	y = 4370.56 x + 675.12	0.9999	-27.37	1
	pickling cowpea	y = 4490.29 x + 532.11	0.9998	-25.38	1
	stir-frying cowpea	y = 4388.09 x - 549.12	0.9997	-27.08	1

Table S4. Residue levels of FLU and TRI and their metabolites FLB and TRA in cucumbers and cowpeas for the different processing operations.

Processing	FLU in cucumbers		FLU in cowpeas		TRI in cucumbers		TRI in cowpeas		FLB in cowpeas		TRA in cucumbers		TRA in cowpeas	
	Residue (μg/kg)	Removal (%)	Residue (μg/kg)	Removal (%)	Residue (μg/kg)	Removal (%)	Residue (μg/kg)	Removal (%)	Residue (μg/kg)	Removal (%)	Residue (μg/kg)	Removal (%)	Residue (μg/kg)	Removal (%)
**Before processing**	287.08±13.51	-	2477.05±19.56	-	235.66±7.01	-	2205.03±186.80	-	5.61±0.22	-	20.51±1.47	-	106.13±6.10	-
**Peeling**		-	-	-		-	-	-	-	-		-	-	-
Skins	581.07±7.21	-	-	-	457.95±20.12	-	-	-	-	-	42.54±1.22	-	-	-
Pulps	85.95±6.33	70.06	-	-	55.61±3.50	76.40	-	-	-	-	2.71±0.28	86.80	-	-
**Washing**														
1 min	221.10±4.27	22.98	1518.01±16.01	38.72	213.25±9.08	9.51	2138.66±125.59	3.01	3.28±0.22	41.59	22.69±1.33	-	80.19±7.47	24.44
3 min	159.15±8.57	44.56	1434.27±21.68	42.10	121.71±2.50	48.35	1528.29±52.97	30.69	3.81±0.51	32.16	18.45±1.63	10.07	56.67±1.47	46.61
5 min	139.27±8.81	51.49	1166.29±61.53	52.92	93.33±5.68	60.39	1482.78±15.25	32.75	3.04±0.10	45.81	14.53±0.68	29.15	47.8±4.82	54.96
7 min	134.55±1.72	53.13	1012.15±7.92	59.14	83.74±2.19	64.47	1537.56±77.51	30.27	4.01±0.52	28.52	13.80±0.30	32.74	45.77±3.29	56.88
10 min	108.20±5.87	62.31	832.32±15.85	66.40	70.68±2.92	70.01	951.66±67.43	56.84	2.30±0.31	58.95	12.81±0.73	37.54	40.96±1.45	61.41
**Stir-frying**														
1 min	84.44±0.51	70.59	1521.52±15.48	38.58	29.18±0.89	87.62	1205.64±18.58	45.32	4.82±0.29	14.10	9.39±0.05	54.22	22.53±2.70	78.77
3 min	99.00±1.89	65.52	1520.15±30.1	38.63	29.97±1.59	87.28	1272.19±	42.30	5.18±0.14	7.74	6.91±0.11	66.30	29.54±0.86	72.16

			1				28.01							
5 min	129.71±5.01	54.82	1608.19±31.01	35.08	41.92±3.89	82.21	1596.99±123.14	27.57	5.50±0.43	1.89	8.39±0.24	59.08	28.82±0.59	72.85
7 min	148.97±7.18	48.11	1714.46±61.58	30.79	64.76±2.88	72.52	1597.43±86.05	27.56	6.58±0.13	-	7.25±0.25	64.66	34.92±0.50	67.10
10 min	167.70±5.98	41.59	1832.05±159.48	26.04	110.27±14.09	53.21	1692.05±56.95	23.26	6.91±0.48	-	5.95±0.53	70.98	41.16±3.81	61.21
**Boiling**														
1 min	252.96±3.36	11.89	1464.72±14.68	40.87	196.16±23.33	16.76	1710.94±86.83	22.41	4.68±0.12	16.61	6.31±0.19	69.24	35.32±3.54	66.72
3 min	213.45±0.76	25.65	1164.14±17.14	53.00	134.47±10.52	42.94	1420.99±33.31	35.56	5.11±0.19	8.83	10.77±0.14	47.50	31.63±2.02	70.20
5 min	195.14±6.09	32.03	1058.60±49.84	57.26	152.28±9.17	35.38	1373.98±103.03	37.69	5.65±0.16	-	8.91±0.25	56.54	27.34±1.88	74.24
7 min	195.29±2.26	31.97	1150.24±49.32	53.56	110.06±1.47	53.30	1000.93±27.33	54.61	5.84±0.24	-	8.41±0.34	58.99	23.54±1.04	77.82
10 min	162.47±4.29	43.41	1147.27±103.99	53.68	100.69±7.09	57.27	782.83±82.82	64.50	4.98±0.43	11.28	9.01±0.14	56.06	20.44±0.31	80.74
**Pickling**														
2h	235.56±3.58	17.95	1281.28±26.31	48.27	175.19±5.00	25.66	1546.73±17.65	29.85	3.08±0.04	45.03	32.65±0.28	-	143.2±1.21	-
1 d	128.29±3.06	55.31	1201.20±34.55	51.51	110.14±0.91	53.26	1276.99±36.64	42.09	2.18±0.15	61.13	29.68±0.47	-	171.72±2.04	-
3 d	132.12±0.97	53.98	1243.09±7.59	49.82	116.45±2.68	50.59	1237.72±71.73	43.87	1.41±0.06	74.84	28.50±0.49	-	219.78±5.94	-
5 d	135.16±3.86	52.92	1249.29±10.5	49.57	115.20±5.59	51.11	1211.02±	45.08	<LOQ	-	35.06±1.07	-	267.48±9.17	-

			0				97.35							
7 d	144.11±1.26	49.80	1735.08±28.83	29.95	118.80±2.29	49.59	1595.62±124.66	27.64	<LOQ	-	47.96±0.31	-	479.86±21.43	-
14 d	153.76± 5.75	46.44	1844.20±144.63	25.55	114.82±4.50	51.28	1901.36±122.72	13.77	<LOQ	-	56.05±3.47	-	574.15±24.33	-

Table S5. Total FLU and TRI residues (expressed as FLU_{sum} and TRI_{sum}) at different sampling intervals and MRLs for different countries.

	Time	Total residues (μg/kg)			MRLs (μg/kg)					
		Average	STMR	HR	China	CAC	US	EU	Japan	Korea
**FLU in cucumbers**										
	2 h	106.15	106.34	108.45						
	1 d	107.65	107.44	110.40						
1st spraying	3 d	55.30	55.13	57.44						
	5 d	54.79	54.85	56.07						
	7 d	21.96	22.14	22.48						
	2 h	226.41	227.06	229.63						
	1 d	195.63	195.91	206.12						
2nd spraying	3 d	197.63	197.92	221.05	500	500	600	600	600	1000
	5 d	172.60	171.98	182.55						
	7 d	120.85	120.77	123.75						
	2 h	215.19	214.73	218.55						
	1 d	248.17	250.14	253.21						
3rd spraying	3 d	209.94	209.79	214.60						
	5 d	152.52	153.40	154.02						
	7 d	157.74	157.61	159.01						
**FLU in cowpeas**										
	2 h	1341.60	1343.75	1396.01						
	1 d	1216.37	1214.13	1251.53						
1st spraying	3 d	1180.10	1172.05	1230.61	1000	1000	4000	3000	-	-
	5 d	1050.42	1051.87	1055.63						
	7 d	878.55	881.55	902.73						

	2 h	2343.05	2349.95	2355.32						
	1 d	2144.15	2145.02	2182.40						
2nd spraying	3 d	2134.03	2125.09	2175.67						
	5 d	2132.91	2113.35	2195.01						
	7 d	1631.10	1631.41	1638.99						
	2 h	3260.36	3260.85	3281.96						
	1 d	2232.33	2217.92	2329.79						
3rd spraying	3 d	2214.06	2216.17	2249.02						
	5 d	2168.87	2161.69	2212.06						
	7 d	2113.08	2104.89	2170.49						
**TRI in cucumbers**										
	2 h	113.69	113.34	121.99						
	1 d	118.10	117.69	127.58						
1st spraying	3 d	65.93	65.75	69.36						
	5 d	52.34	52.74	53.17						
	7 d	20.44	20.32	22.04						
	2 h	230.31	231.62	241.82						
	1 d	168.31	167.90	171.57						
2nd spraying	3 d	194.29	191.80	204.53	300	300	500	300	700	500
	5 d	92.06	91.94	94.48						
	7 d	108.61	107.19	115.40						
	2 h	245.48	245.73	254.97						
	1 d	259.63	258.67	277.61						
3rd spraying	3 d	214.59	215.53	238.22						
	5 d	152.94	153.29	161.45						
	7 d	135.98	134.03	149.50						

**TRI in cowpeas**										
	2 h	1592.24	1559.61	1796.70						
	1 d	1402.04	1398.38	1435.80						
1st spraying	3 d	1251.89	1263.32	1272.13						
	5 d	1288.79	1292.62	1358.86						
	7 d	1168.93	1141.32	1342.91						
	2 h	2679.22	2677.12	2806.77						
	1 d	2257.34	2249.29	2332.58						
2nd spraying	3 d	2245.24	2238.47	2366.34	-	-	1500	1500	-	-
	5 d	2251.35	2245.16	2315.84						
	7 d	2004.10	2008.59	2077.21						
	2 h	3613.37	3620.67	3695.32						
	1 d	2725.25	2717.57	2817.48						
3rd spraying	3 d	2479.15	2485.29	2521.95						
	5 d	2326.64	2320.67	2372.12						
	7 d	2176.81	2155.65	2285.72						
